# Gambling and Substance Use: Early Evidence From Sports Betting Laws

**DOI:** 10.1002/hec.70088

**Published:** 2026-03-11

**Authors:** Kabir Dasgupta, Keshar Ghimire

**Affiliations:** ^1^ Division of Consumer & Community Affairs Federal Reserve Board Washington DC USA; ^2^ Business and Economics Department University of Cincinnati Blue Ash Ohio USA

**Keywords:** drinking, gambling, smoking, sports betting laws, substance use

## Abstract

Previous research documents a strong association between gambling and substance use, suggesting that these seemingly distinct behaviors may share similar environmental, neurobiological, and genetic causes. However, there is a dearth of credible empirical evidence on whether gambling has a causal impact on substance use or vice versa. This paper estimates the impact of gambling on substance use making use of the rapid roll‐out of sports betting laws across US states. Using data from the Behavioral Risk Factor Surveillance System and a difference‐in‐difference (DID) estimation strategy to assess the impact of legalizing sports betting on smoking and drinking behavior among adults, we find that the legalization of online sports betting has increased binge drinking frequency‐at the intensive margin among young men by approximately 10 percent, but find no discernible impact on smoking. The results are consistent across traditional two‐way fixed effects models as well as more recently developed DID methods designed for staggered treatment adoption.

## 1 Introduction

The rapid expansion of legalized sports betting across the United States after the 2018 Supreme Court ruling in *Murphy v. National Collegiate Athletic Association (2018)* has raised concerns about its potential impact on public health, on aspects including but not limited to problem gambling, mental health, and substance use disorders (Humphreys and Ruseski 2024; Badji et al. 2023; Couture et al. 2024). Although previous research has established a correlation between gambling and substance use disorders, the causal relationship remains underexplored. We address this gap in the literature by examining whether the legalization of sports betting influences the consumption of tobacco and alcohol.

Policy discussions surrounding sports betting legalization have increasingly emphasized that potential harms may not be evenly distributed across the population (J. Croup 2025). Specifically, concerns have been raised about the rapid expansion of online sports betting, which lowers participation costs and disproportionately attracts younger male users, a group that also exhibits higher baseline rates of risky drinking behaviors. Motivated by this discussion, we examine not only population‐wide effects but also whether legalization affects substance use intensity within groups most exposed to sports betting opportunities.

We utilize data from the Centers for Disease Control and Prevention's (CDC) Behavioral Risk Factor Surveillance System (BRFSS) to compare substance use across US states that have legalized sports betting to those that have not. We employ a difference‐in‐difference strategy to identify causal mechanisms. Given that sports betting laws were implemented in a staggered fashion, we employ Callaway and Sant’Anna's (2021) difference‐in‐differences technique to rule out any potential bias resulting from heterogeneous treatment effects across cohorts.

We find that legalization of sports betting ‐ access to online betting in particular ‐ increased binge drinking, along the intensive margin, among young men aged 35 years and under. These results are robust to variations in specifications, and modeling techniques and align well with conclusions from recent survey‐based studies such as Grubbs and Kraus (2024). One caveat for our results is that binge‐drinking may have been affected by the COVID‐19 pandemic (Weerakoon et al. 2021).^1^ However, as long as this effect was comparable across treatment and control states, our identification strategy should yield credible estimates.

## 2 Literature

Research has for long established the nexus between gambling and substance use. Substance abusers experience pathological gambling at a much higher rate than general population and individuals seeking treatment for gambling issues often exhibit high levels of substance dependence (Petry 2007). Similarly, problem gambling and alcohol/tobacco use are strongly correlated, with the association varying across demographic groups. For example, Barnes et al. (2015) find that problem gambling was most prevalent among economically vulnerable black men with alcohol and tobacco dependence. Other studies report high prevalence of gambling behavior among young males under 35 (Griffiths et al. 2009).

Scholes‐Balog and Hemphill (2012) suggest that online gamblers are at a greater risk of substance abuse and mental disorders relative to non‐online gamblers. More recently using Spanish data on gambling and consumption choices, Díaz and Pérez (2021) find that people with higher levels of tobacco and alcohol use are likely to spend more on gambling activities. The authors are agnostic about the direction of causality. Using longitudinal data from Australia, Badji et al. (2023) explore economic, health and behavioral implications of access to gambling venues. The authors find that proximity to gambling venues ‐ which is greatly influenced in the context of online access ‐ is associated with more gambling, less happiness, more financial hardships and more mental health problems. Notably Badji et al. (2023) also find the largest effects for young males in low‐income jobs.

In the US, the legalization and expansion of sports betting have prompted concerns regarding potential public health implications, including gambling addiction, mental health, and substance abuse. As such, a growing number of empirical studies have been focusing on the wellbeing implications of sports betting laws (SBL). For example, Couture et al. (2024) evaluate these state‐level SBLs and conclude, based on data from the BRFSS, that “legalized gambling leads to a 5.5% points decrease ‐ over 11% ‐ in the likelihood of poor mental health for men between 18 and 24, but a 3.8% point increase ‐ nearly 10% ‐ for men between 30 and 34.” Humphreys and Ruseski (2024) also focus on sports betting legalization and mental health data from BRFSS to find that access to sports betting hampers mental health along several dimensions including having ‘bad days’ and ‘depressive disorder’. In contrast, however, Humphreys et al. (2021) using Canadian data find that recreational gambling has positive impact on life satisfaction, reduced stress levels, and improved self‐assessed health status.

On a related project, Hollenbeck et al. (2024) examine the financial consequences of SBLs and find that “overall consumers' financial health is modestly deteriorating as the average credit score in states that legalize sports gambling decreases by roughly 0.3%.” In the same vein, Baker et al. (2024) utilize extensive household transaction level data to estimate the impact of SBLs on household investment, spending and debt management decisions to find a series of negative outcomes including reduced savings, lower investments, higher credit debt balances and higher overdraft.

Overall, several studies have documented a strong association between gambling and substance use, and many have recently zeroed in on the impact of US SBLs on mental health and financial consequences. We extend the literature by estimating the causal impact of expanded access to gambling on smoking and drinking behavior. Since increased consumption of alcohol and tobacco is clearly associated with several negative outcomes, we provide an important insight into a public health challenge that arises as a spillover effect of SBLs.^2^

## 3 Data and Empirical Strategy

Our main source of information on Sports Betting Laws is the website of the American Gaming Association which provides a comprehensive list of sports betting provisions across US states including information on retail and/or online access with dates of legalization and implementation.^3^ Implementation years as coded in our sample are shown in Table A2.^4^ About 19% of our sample has online access and close to 29% of the sample has some form of access to sports betting. Our outcome variables and individual level covariates come from the Centers for Disease Control and Prevention's (CDC) BRFSS.^5^ The BRFSS provides detailed health and demographic information of a nationally representative sample of adults from across all 50 states and the District of Columbia. Because every state except Nevada legalized sports betting in or after 2018, we utilize surveys from 2016 through 2023 to ensure the inclusion of sufficient pre‐treatment observations in our sample.

To investigate the causal impact of sports betting legalization on substance use, we employ a DID estimation strategy, leveraging the staggered roll‐out of sports betting laws across U.S. states. For our baseline two‐way fixed effects (TWFE) model, we estimate:

(1) $$\begin{array}{c} Y_{\mathrm{ist}}=\alpha +\rho .SBL_{\mathrm{ist}}+X_{ist}^{\prime }.\beta +Z_{ist}^{\prime }.\delta +\Omega_{s}+\lambda_{t}+\upsilon_{\mathrm{ist}} \end{array}$$

where $Y_{\mathrm{ist}}$ represents the substance use behavior of individual i in state s at time t. The variable $SBL_{\mathrm{ist}}$ is denoted by a 0‐1 binary indicator that switches to 1 from the year when state s has legalized sports betting. The parameter, ρ represents the TWFE estimator of the effect of sports betting legalization on individuals' substance use behavior. For further precision of our estimates, we control for individual‐level demographic characteristics including, age, sex, race, ethnicity, marital status, and educational attainment. We also control for relevant state‐specific time‐variant characteristics including measures of state economic performance such as unemployment rate, gross state product and average personal income, and political affiliation of the Governor as a proxy for socio‐political environment. We extract this information from the National Welfare Data available from the University of Kentucky Center for Poverty Research (UKCPR).^6^ Additionally, we control for existing recreational marijuana laws (RML), beer taxes and tobacco taxes to account for broader regulatory environment with which the SBLs are interacting.^7^ Inclusion of such state‐specific measures addresses potential confounding influences from unobserved heterogeneities that could jeopardize the causal interpretation of estimated parameter of interest ρ. For instance, states that have stricter regulations against substance use may have a similar stance for legalizing online sports betting. Finally, we also control for state‐ and time‐fixed effects, as represented by $\lambda_{t}$ and $\Omega_{s}$. Unless otherwise stated, in all estimations, we apply BRFSS final sampling weights to account for the complex survey design and ensure representativeness of the adult population within each state‐year. Standard errors are adjusted for clustering at the state level.

The staggered setting in Equation (1) can be easily extended to the Callaway and Sant’Anna's (2021) DID (or CSDID) framework. Broadly, the CSDID methodology separately estimates TWFE type regressions by comparing outcomes of a treatment group (states that legalized betting at the same time) to that of ‘not‐yet‐treated’ or ‘never‐treated’ states, and then averaging those group‐specific effects to compute an aggregate point estimate of the effect of SBLs.

## 4 Results

**FIGURE A1:**
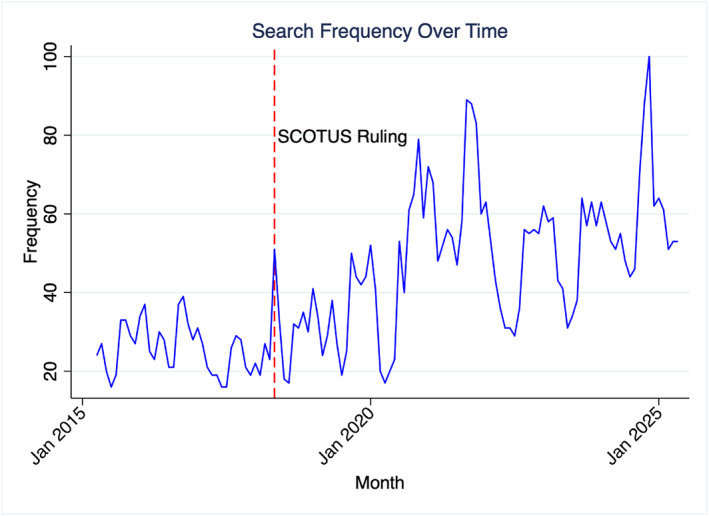
Interest in sports betting over time: Google search trends. Numbers on *y*‐axis represent search interest relative to the highest point on the chart for the given region and time. A value of 100 is the peak popularity for the term. A value of 50 means that the term is half as popular. A score of 0 means there was not enough data for this term.

**FIGURE A2:**
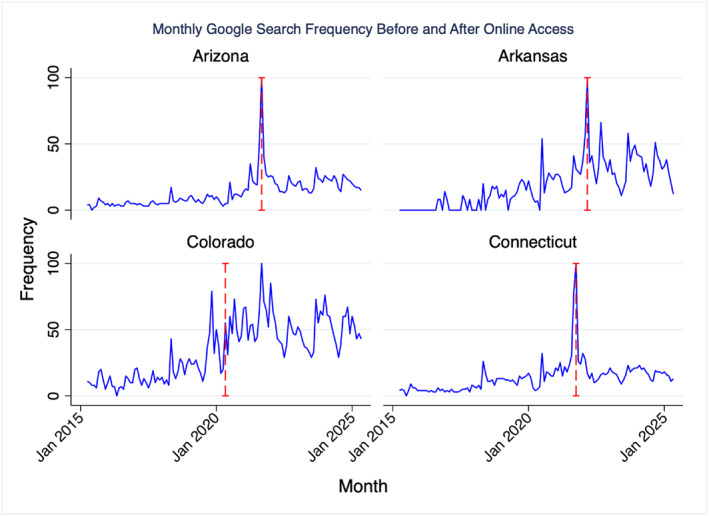
Interest in sports betting over time: Google search trends by individual states. Numbers on *y*‐axis represent search interest relative to the highest point on the chart for the given region and time. A value of 100 is the peak popularity for the term. A value of 50 means that the term is half as popular. A score of 0 means there was not enough data for this term. Dashed vertical lines mark the beginning of online access to betting.

**FIGURE A3:**
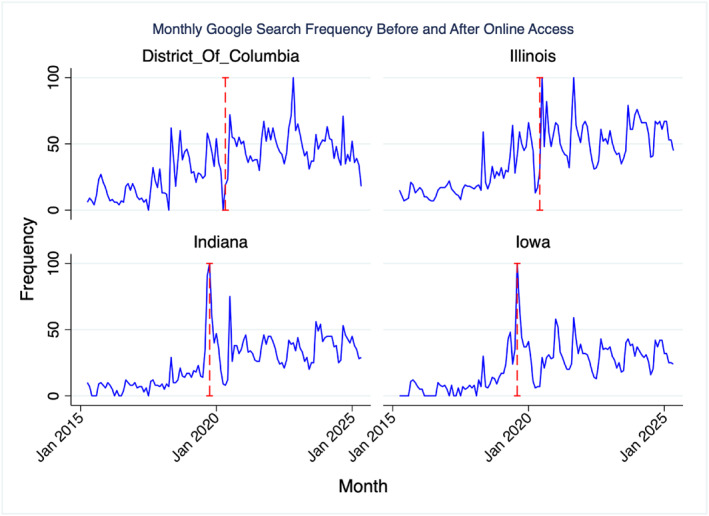
Interest in sports betting over time: Google search trends by individual states. Numbers on *y*‐axis represent search interest relative to the highest point on the chart for the given region and time. A value of 100 is the peak popularity for the term. A value of 50 means that the term is half as popular. A score of 0 means there was not enough data for this term. Dashed vertical lines mark the beginning of online access to betting.

**FIGURE A4:**
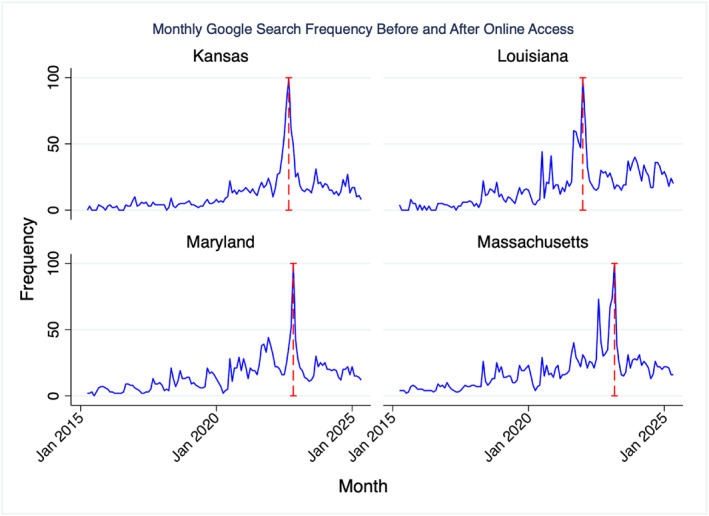
Interest in sports betting over time: Google search trends by individual states. Numbers on *y*‐axis represent search interest relative to the highest point on the chart for the given region and time. A value of 100 is the peak popularity for the term. A value of 50 means that the term is half as popular. A score of 0 means there was not enough data for this term. Dashed vertical lines mark the beginning of online access to betting.

**FIGURE A5:**
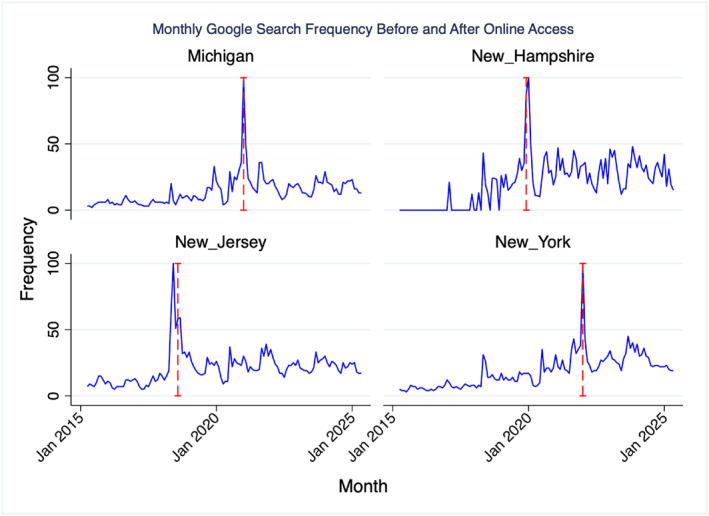
Interest in sports betting over time: Google search trends by individual states. Numbers on *y*‐axis represent search interest relative to the highest point on the chart for the given region and time. A value of 100 is the peak popularity for the term. A value of 50 means that the term is half as popular. A score of 0 means there was not enough data for this term. Dashed vertical lines mark the beginning of online access to betting.

**FIGURE A6:**
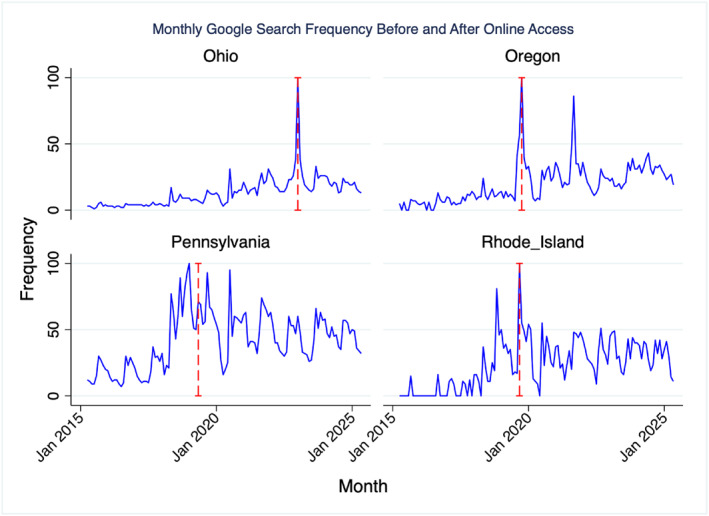
Interest in sports betting over time: Google search trends by individual states. Numbers on *y*‐axis represent search interest relative to the highest point on the chart for the given region and time. A value of 100 is the peak popularity for the term. A value of 50 means that the term is half as popular. A score of 0 means there was not enough data for this term. Dashed vertical lines mark the beginning of online access to betting.

**FIGURE A7:**
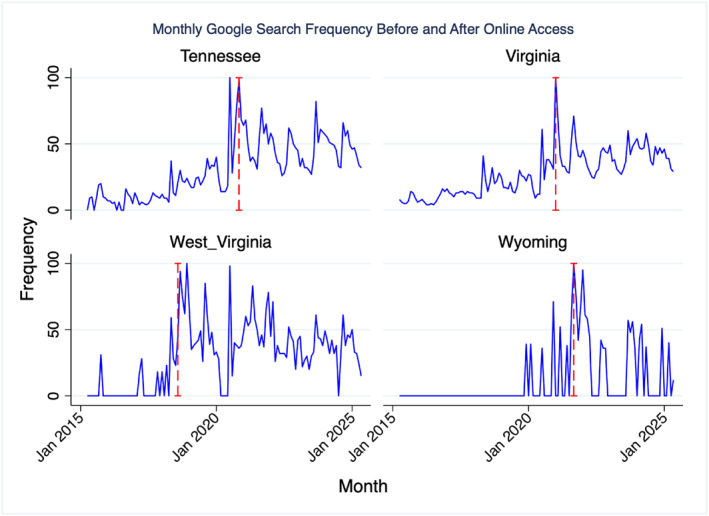
Interest in sports betting over time: Google search trends by individual states. Numbers on *y*‐axis represent search interest relative to the highest point on the chart for the given region and time. A value of 100 is the peak popularity for the term. A value of 50 means that the term is half as popular. A score of 0 means there was not enough data for this term. Dashed vertical lines mark the beginning of online access to betting.

**FIGURE A8:**
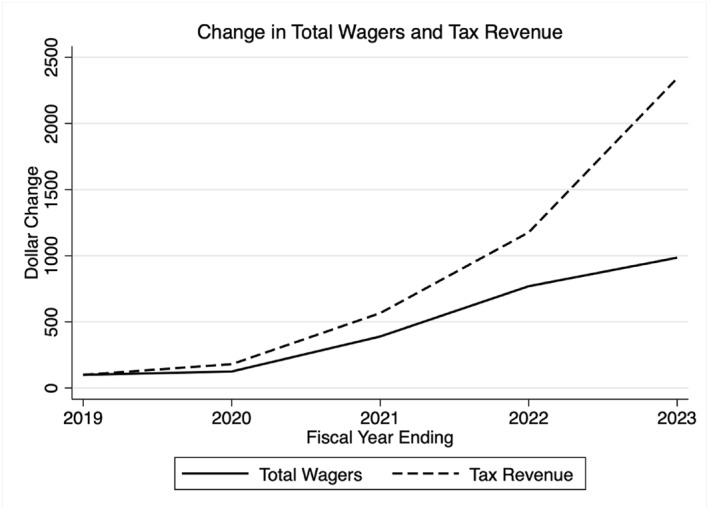
Change in total wagers and state tax revenue from sports betting over years. Numbers on *y*‐axis are indexed such that revenue in FY2019 is 100 and the following years are relative to 2019. Taxfoundation.org.

In absence of individual level data to directly test the ‘first stage’ effect of whether SBLs increased gambling, we examine the effect of laws on interest in sports betting as measured by changes in searches on Google. Interest in sports betting has drastically increased after the 2018 Supreme court decision that declared the Professional and Amateur Sports Protection Act (PASPA) unconstitutional. We see this spike in the Google search trends data on the topic of ‘sports betting’ when comparing period before and after 2018. Figure A1 shows that US google searches on the topic have almost doubled in the aftermath of the court ruling. We examine state‐specific trends in figures A2, A3, A4, A5, A6, A7, A8 and observe, not surprisingly, that for most of the states that implemented online access, there was a notable spike in online interest around the time of the implementation. While these trends do not directly track sports gambling activity, they complement the reports that states with SBLs are experiencing significant surge in sports betting revenue (Simon 2025) and that almost all of that growth is coming from online sports betting (American Gaming Association 2025). Figure A8 shows the dramatic evolution of wagers and revenues in the post 2018 era.

We begin analysis of our BRFSS sample by comparing the summary statistics across treated and control states presented in Table 1.^8^ The statistics for treated states are based on observations before treatment. We distinguish between ‘any treated’ and ‘online treated’ for comparison across groups within each treatment type. We see that treated and controls states are fairly comparable in terms of outcomes. In terms of covariate balance across treated and control states, we observe similar comparability except in terms of race (treated states have a greater share of White population, while control states have a higher share of Black population), political leaning (treated states are more likely to have a Democrat Governor), and tobacco/beer taxes (treated states have higher tobacco taxes but lower beer taxes).

**TABLE 1:** Summary statistics for outcomes and controls.

	(1)	(2)	(3)	(4)
	Any control	Any treated	Online control	Online treated
	Mean	Mean	Mean	Mean
Sports betting laws:				
Any law	0.0000	0.0000	0.0621	0.0974
Online law	0.0000	0.0000	0.0000	0.0000
Outcome variables:				
Smoked>100 cigs in lifetime (u)	0.4117	0.4335	0.4147	0.4328
Current smoker (c)	0.3283	0.3362	0.3320	0.3316
Current smoker (u)	0.1349	0.1454	0.1344	0.1454
Any smoke‐free tobacco (u)	0.0348	0.0328	0.0361	0.0306
Days drank in last 30 days (u)	4.9093	5.1746	4.9211	5.1785
Current drinker (u)	0.4820	0.5256	0.4963	0.5205
Average drink per day (c)	2.2954	2.1677	2.3043	2.1486
Average drink per day (u)	1.1064	1.1393	1.1200	1.1291
Binge days in last 30 days (c)	4.7978	4.4679	4.7359	4.5173
Any binge in last 30 days (u)	0.1320	0.1333	0.1332	0.1312
Covariates:				
Marital status	0.5250	0.5165	0.5253	0.5111
High school	0.2667	0.2698	0.2687	0.2677
Some college	0.2845	0.2725	0.2861	0.2685
College degree	0.3741	0.3906	0.3719	0.3963
Female	0.5454	0.5546	0.5447	0.5546
Age	54.7437	55.3910	54.8138	55.4989
White	0.6610	0.7728	0.6635	0.7673
Black	0.0637	0.0807	0.0657	0.0797
Hispanic	0.0887	0.0635	0.0807	0.0706
Unemployment rate	4.1199	4.4747	4.0909	4.7016
GSP (Billions)	594.10	460.90	542.53	563.88
PI (Billions)	529.74	396.48	483.33	479.88
Poverty rate	11.2631	10.9109	11.3608	10.8946
Governor is democrat	0.2837	0.4306	0.3090	0.4710
Tobacco tax (cents/pack)	157.73	197.04	153.11	220.82
RML	0.1725	0.1720	0.1460	0.1844
Beer tax ($/gal)	0.4124	0.2077	0.3925	0.1934
Observations†	1,255,537	1,113,873	1,482,884	1,084,175

*Note:* Data from BRFSS and UKCPR, 2016‐2023. Dollar values are in 2020 dollars. Calculations for treated states are for pre‐treatment period. Variables that have (c) represent conditional measures. Variables that have (u) denote undconditional measures. † The observations shown correspond to the maximum number of observations available within each treatment and control group. Due to BRFSS questionnaire pattern, the effective sample size varies across outcome variables, and not all respondents contribute to every measure. See Table A1 for more detailed summary stats on each variable.

Abbreviations: GSP: Gross State Product. PI: Personal Income.

In Table 2, we present DID estimates for the impact of SBL on several measures of substance use. Panel A includes results from TWFE estimation and Panel B includes aggregated estimates from the CSDID model. In each panel, we present effects of having any kind of sports betting legalization (i.e., online or retail) and that of having online sports betting legalization. Our preferred estimations are those in panel B as these are robust to heterogeneous treatment effects which we cannot rule out in the current setting.

**TABLE 2:** Impact of sports betting laws on substance use: Full sample.

	(1)	(2)	(3)	(4)	(5)	(6)	(7)	(8)	(9)	(10)
	Smoked>100 Cigs. (u)	Current Smoker (c)	Current Smoker (u)	Smoke‐free Tobacco (u)	Days Drank (u)	Current Drinker (u)	Drinks per day (c)	Drinks per days (u)	Binge days (c)	Any Binge (u)
TWFE:										
Any law	−0.0032	−0.0016	−0.0018	0.0001	0.0343	−0.0031	0.0437*	0.0161	0.0229	−0.0021
	(0.0024)	(0.0025)	(0.0016)	(0.0010)	(0.0561)	(0.0060)	(0.0234)	(0.0237)	(0.0720)	(0.0033)
Online law	−0.0020	−0.0023	−0.0018	0.0001	0.0580	−0.0080	0.0425*	−0.0026	0.0475	−0.0056
	(0.0025)	(0.0028)	(0.0016)	(0.0010)	(0.0575)	(0.0064)	(0.0234)	(0.0261)	(0.0647)	(0.0035)
*N*	2,775,417	1,170,193	2,775,417	2,782,613	2,733,168	2,733,168	1,382,216	2,733,168	363,721	2,733,168
CSDID:										
Any law	−0.0036	−0.0022	−0.0034	−0.0002	0.0202	−0.0014	0.0036	−0.0018	0.0805	−0.0049
	(0.0101)	(0.0111)	(0.0080)	(0.0037)	(0.1246)	(0.0124)	(0.0325)	(0.0348)	(0.1299)	(0.0049)
Online law	0.0012	0.0016	0.0006	0.0001	−0.0142	−0.0032	0.0280	0.0085	0.0695	−0.0012
	(0.0079)	(0.0090)	(0.0063)	(0.0028)	(0.1001)	(0.0147)	(0.0316)	(0.0318)	(0.1103)	(0.0052)
Observations	3,197,776	1,334,150	3,206,362	3,204,866	3,147,571	3,147,571	1,599,694	3,147,571	419,884	3,147,571

*Note:* Standard errors adjusted for clustering around states in parentheses. Coefficients are from weighted regressions with individual level controls (age, sex, marital status, race, ethnicity, and education), state level controls (unemployment rate, gross domestic product, personal income, poverty rate, and governor's party affiliation), and state & year fixed effects. Data is BRFSS 2016‐2023.

^*^ (*p* < 0.10).

**(*p* < 0.05).

***(*p* < 0.01).

Given the structure of the BRFSS questionnaire, we examine the impact of sports betting laws on several measures of tobacco and alcohol consumption that differ in whether they are defined for the full sample or for specific subsamples of respondents. For smoking‐related outcomes, all BRFSS respondents are asked whether they have smoked more than 100 cigarettes in their lifetime. Those who answer affirmatively are then asked a follow‐up question about their current smoking behavior. Therefore, we construct three smoking measures. First, ‘smoked>100 cigarettes’ is an indicator equal to one if the respondent reports having smoked more than 100 cigarettes in their lifetime. Second, ‘current smoker (conditional)’ is a binary indicator for current smoking status, defined only for respondents who report having smoked more than 100 cigarettes; this variable is therefore conditional on ever having been a smoker. Third, we construct an unconditional measure of current smoking status, ‘current smoker (unconditional)’, by coding all respondents who do not report current smoking‐including those who report having smoked fewer than 100 cigarettes in their lifetime‐as zero. Finally, all respondents are asked whether they currently use smokefree tobacco; therefore, ‘smokefree tobacco use’ is an unconditional indicator defined for the full sample.

We next construct seven measures related to alcohol consumption. All respondents are asked about the number of days they consumed alcohol in the past 30 days; we use this information to construct ‘days drank’, a continuous variable measuring drinking frequency. Only respondents who report at least one drinking episode in the past 30 days (hereafter, “drinkers”) are asked follow‐up questions about drinking intensity. Among this subsample, we construct ‘drinks per day (conditional)’, which measures the average number of drinks consumed per day. We also construct an unconditional counterpart, ‘drinks per day (unconditional)’, by assigning a value of zero to nondrinkers and averaging across the full sample. In addition, we define ‘any drinking’ as a binary indicator equal to one for respondents who report drinking at least once in the past 30 days and zero otherwise. Finally, we construct two measures related to binge drinking. The BRFSS asks all drinkers how many days in the past 30 days they engaged in binge drinking.^9^ Using this information, we define ‘binge days’ as the number of binge‐drinking days among respondents who report at least one binge episode, which captures the intensive margin of binge drinking. We also construct ‘any binge (unconditional)’ which is a binary indicator equal to one for any respondent who reports at least one binge‐drinking day and zero for all others, including nondrinkers.

All coefficients, except one, presented in Table 2 indicate null effects. We see that, per TWFE estimates, SBLs have a positive impact on average number of drinks per day (conditional measure). However these effects disappear when applying the CSDID method.

As previously discussed, several previous studies indicate that gambling is more concentrated among young men aged 35 and under. Therefore, we study the results from TWFE and CSDID estimation for sub‐samples of young men (Table 3) and young women (Table 4). In Table 3, our CSDID specification indicates that legalizing online sports betting prompts a statistically significant increase in frequency of binge drinking. Specifically, we observe an average treatment effect (ATT) of 0.46 on binge days for binge‐drinkers. This estimate represents increase of approximately 10% relative to the average of 4.6 binge days for young men in treated states in the pretreatment period. The coefficient on ‘any law’ is positive but statistically insignificant. We do not see any statistically significant impact on the unconditional binge probability suggesting that the increase is on the intensive margin, not extensive. In regards to young women, CSDID estimates in Table 4 indicate null effects of SBLs on their tobacco and alcohol consumption.

**TABLE 3:** Impact of sports betting laws on substance use: Young men.

	(1)	(2)	(3)	(4)	(5)	(6)	(7)	(8)	(9)	(10)
	Smoked>100 Cigs. (u)	Current Smoker (c)	Current Smoker (u)	Smoke‐free Tobacco (u)	Days Drank (u)	Current Drinker (u)	Drinks per day (c)	Drinks per days (u)	Binge days (c)	Any Binge (u)
TWFE:										
Any law	−0.0054	0.0137	0.0010	0.0003	0.1275	−0.0075	0.1549***	0.0662	0.2836*	−0.0049
	(0.0052)	(0.0087)	(0.0046)	(0.0034)	(0.0947)	(0.0085)	(0.0551)	(0.0487)	(0.1461)	(0.0090)
Online law	0.0024	0.0137	0.0038	0.0011	0.1709	−0.0102	0.0639	0.0101	0.4390***	−0.0126
	(0.0082)	(0.0097)	(0.0039)	(0.0032)	(0.1256)	(0.0117)	(0.0752)	(0.0721)	(0.1413)	(0.0112)
*N*	251,763	83,415	251,763	252,216	247,136	247,136	152,148	247,136	76,742	247,136
CSDID:										
Any law	0.0013	−0.0116	−0.0082	−0.0046	−0.0051	−0.0016	−0.0350	−0.0251	0.1442	−0.0139
	(0.0105)	(0.0169)	(0.009)	(0.0082)	(0.1154)	(0.0112)	(0.0731)	(0.0561)	(0.1204)	(0.0085)
Online law	−0.0006	−0.0109	−0.0041	0.0049	0.1608	0.0025	0.0395	0.0314	0.4622***	−0.0048
	(0.0082)	(0.0169)	(0.0085)	(0.0092)	(0.1741)	(0.0113)	(0.0802)	(0.0585)	(0.1775)	(0.0095)
Observations	292,571	94,333	292,160	292,683	286,741	286,741	176,252	286,741	88,288	286,741

*Note:* Standard errors adjusted for clustering around states in parentheses. Coefficients are from weighted regressions with individual level controls (age, marital status, race, ethnicity, and education), state level controls (unemployment rate, gross domestic product, personal income, poverty rate, and governor's party affiliation), and state & year fixed effects. Data is BRFSS 2016‐2023.

*(*p* < 0.10).

**(*p* < 0.05).

^***^ (*p* < 0.01).

**TABLE 4:** Impact of sports betting laws on substance use: Young women.

	(1)	(2)	(3)	(4)	(5)	(6)	(7)	(8)	(9)	(10)
	Smoked>100 Cigs. (u)	Current Smoker (c)	Current Smoker (u)	Smoke‐free Tobacco (u)	Days Drank (u)	Current Drinker (u)	Drinks per day (c)	Drinks per days (u)	Binge days (c)	Any Binge (u)
TWFE:										
Any law	−0.0020	−0.0030	−0.0009	0.0019	−0.0068	0.0042	0.0288	0.0265	−0.0583	−0.0007
	(0.0044)	(0.0108)	(0.0038)	(0.0012)	(0.0714)	(0.0080)	(0.0340)	(0.0277)	(0.1108)	(0.0052)
Online law	−0.0096**	−0.0187*	−0.0094***	0.0015	−0.0643	−0.0093	0.0274	−0.0056	−0.0397	−0.0034
	(0.0040)	(0.0102)	(0.0033)	(0.0015)	(0.0605)	(0.0067)	(0.0357)	(0.0261)	(0.1197)	(0.0044)
*N*	238,898	61,862	238,898	239,078	234,798	234,798	126,452	234,798	48,308	234,798
CSDID:										
Any law	−0.0018	−0.0174	−0.0088	0.0008	0.0396	0.0068	0.0209	0.0268	−0.0242	−0.0052
	(0.0155)	(0.0192)	(0.0104)	(0.0020)	(0.1267)	(0.0107)	(0.0467)	(0.0376)	(0.1395)	(0.0105)
Online law	0.0118	−0.0156	−0.0013	0.0019	0.1457	0.0137	0.0330	0.0507	0.0017	−0.0023
	(0.0143)	(0.0182)	(0.0091)	(0.0022)	(0.1395)	(0.0139)	(0.0425)	(0.0408)	(0.1631)	(0.0120)
Observations	274,859	68,641	274,859	275,018	269,987	269,987	146,076	269,987	55,809	269,987

*Note:* Standard errors adjusted for clustering around states in parentheses. Coefficients are from weighted regressions with individual level controls (age, marital status, race, ethnicity, and education), state level controls (unemployment rate, gross domestic product, personal income, poverty rate, and governor's party affiliation), and state & year fixed effects. Data is BRFSS 2016‐2023.

^*^ (*p* < 0.10).

^**^ (*p* < 0.05).

^***^ (*p* < 0.01).

**FIGURE 1:**
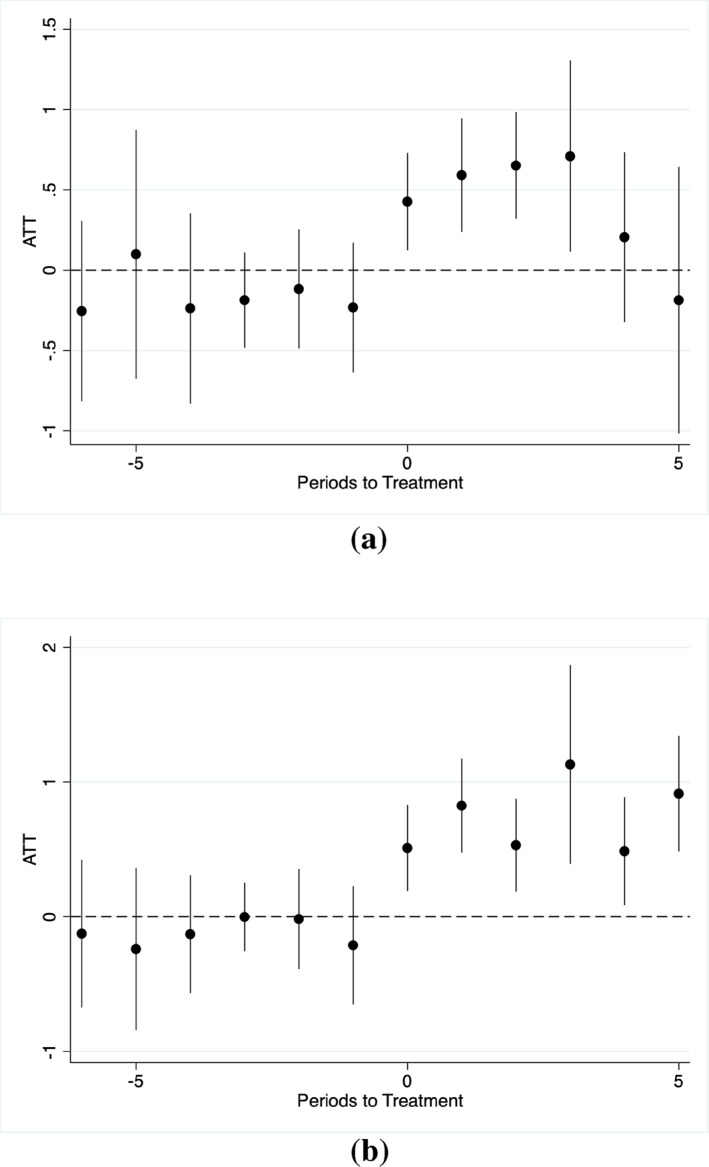
CSDID event study plot for test of parallel trends in binge drinking among young men. (a) Dynamic impact of any sports betting law on binge drinking of young men. (b) Dynamic impact of online sports betting law on binge drinking of young men. Figures show CSDID coefficient estimates (ATTs averaged across treatment cohorts) with 95% confidence intervals on indicators for relative time from policy implementation. Data is BRFSS 2016‐2023.

For credibility of our finding related to binge drinking in young men, we estimate the dynamic treatment effects of the laws and plot estimates in Figure 1. That there are no significant pre‐treatment effects of any law and online law suggests that the assumption of parallel trends is reasonable. We observe positive significant effects in the post‐treatment period in case of both law types. These results are consistent with our finding of positive and significant ATT obtained from CSDID estimation. One interesting difference between any law and online law is that effects of the former intervention type seem to dissipate after certain period of elevation whereas the effects of online access do not fade even after 5 years of access. This contrasting evidence reinforces the potency of online access highlighted in previous research (e.g., see Scholes‐Balog and Hemphill (2012)).

In light of evidence that COVID‐19 may have affected binge drinking, we further test the sensitivity of these findings for young men by limiting sample to pre‐COVID years and present results in the upper panel of Table A3. Results are qualitatively similar to the main results. In line with previous research, our main results indicate that the online access may be stronger relative to any kind of access. We test sensitivity of this finding by restricting the sample to only treated states (any law) and then checking the effect of online law within that sample. The results, presented in the middle panel of Table A3, are consistent.

Next we explore heterogeneity across race, age, marital status and education and present results in Table A4. We see statistically significant increases in binge frequency of young men in the categories of Black, unmarried, and non‐college educated individuals. Focusing on age groups, we see increases in 25–29 and 30–34 year olds but not among 18–24 year olds.^10^

## 5 Discussion and Conclusion

We provide early causal evidence on the relationship between expanded gambling opportunities and substance use behaviors. Exploiting the staggered roll‐out of state‐level sports betting legalization and employing rigorous DID methods, we find that the introduction of online sports betting led to a significant uptick in alcohol misuse among a specific demographic. In particular, the legalization of online sports wagering caused approximately a 10% increase in the frequency of binge drinking among young males (an intensive margin effect). By contrast, our estimates show no meaningful changes in tobacco consumption behavior. Taken together, these findings suggest that the public health impact of SBLs may manifest in targeted ways ‐ notably through elevated alcohol consumption in young males who already are heavy drinkers ‐ rather than as broad‐based increases in all forms of substance use. However, the analysis captures only the initial years following SBLs, so longer‐term effects remain uncertain. In addition, the BRFSS data rely on self‐reported behaviors, which may understate true consumption levels.

Our estimates represent the intent‐to‐treat effects of SBLs and pave way for further investigations using granular consumer data on substance use, especially for the demographic groups like young men who are most affected by these laws. Moreover, the estimated effects likely include any direct effects of gambling (such as drinking while placing bets and/or following game stats) and indirect effects operating through mediating channels such as adverse mental and financial consequences of gambling.^11^ Disentangling relative importance of these pathways can help inform any potential policy intervention.

## Author Contributions

The authors thank Faham Tak for research assistance. The results and opinions expressed in this study reflect the views of the authors and should not be attributed to the Federal Reserve Board or the Federal Reserve System. The authors are solely responsible for all errors committed.

## Funding

The authors have nothing to report.

## Conflicts of Interest

The authors declare no conflicts of interest..

## Data Availability

The data that support the findings of this study are available in Behavioral Risk Factor Surveillance System (BRFSS) at https://www.cdc.gov/brfss/. These data were derived from the following resources available in the public domain: ‐ Centers for Disease Control and Prevention (CDC), BRFSS, https://www.cdc.gov/brfss/data_documentation/index.htm.

## Endnotes

^1^
Weerakoon et al. (2021) find that increase in the time spent at home during the pandemic prompted a rise in the likelihood of binge‐drinking. Our results are qualitatively similar when analyzing a pre‐Covid sample.

^2^
See Dai et al. (2022), Dasgupta et al. (2022), Zhong et al. (2022), and Gorelick (2023) for a quick review of negative outcomes associated with substance use.

^3^
See https://www.americangaming.org/research/state‐of‐play‐map/ for details.

^4^
When a law is implemented prior to July 1 of any year, we code that year as the first year of implementation. Otherwise the following year is coded as the first year of implementation.

^5^
Data is publicly available at https://www.cdc.gov/brfss/data_documentation/index.htm.

^6^
The welfare data is publicly available for download at https://cpr.uky.edu/resources/national‐welfare‐data.

^7^
We obtain the RML information from information from Abouk et al. (2023). The information on beer and tobacco taxes comes from the website of Tax Foundation.

^8^
We present extended summary statistics, including the minimum value, maximum value, and number of observations (N) for each variable in Table A1.

^9^
Binge drinking is defined as consuming four or more drinks for women and five or more drinks for men on a single occasion. One drink is defined as a 12‐ounce beer, 5 ounces of wine, or 1.5 ounces of distilled spirits.

^10^
In unreported regressions we tested the impact of SBLs on underage drinking by limiting sample to 18‐20 and did not find any effect.

^11^
SBLs have previously been shown to adversely impact mental health and financial condition (Couture et al. 2024; Hollenbeck et al. 2024). Because there exists strong evidence for association between financial/mental adversity and substance use (Drake and Mueser 1996; Cotti et al. 2015).

## Appendix

Figure A1, A2, A3, A4, A5, A6, A7, A8

Table A1, A2, A3, A4

**TABLE A1:** Expanded summary statistics for outcomes and controls.

	Any law control states				Any law treated states, (pretreatment)			
	Mean	Min	Max	*N*	Mean	Min	Max	*N*
Sports betting laws								
Any law	0.000	0.000	0.000	1,255,537	0.000	0.000	0.000	1,113,873
Online law	0.000	0.000	0.000	1,255,537	0.000	0.000	0.000	1,113,873
Outcomes								
Smoked>100 cigs in lifetime (u)	0.412	0.000	1.000	1,197,090	0.434	0.000	1.000	1,066,220
Current smoker (c)	0.328	0.000	1.000	491,736	0.336	0.000	1.000	461,171
Current smoker (u)	0.135	0.000	1.000	1,197,090	0.145	0.000	1.000	1,066,220
Any smoke‐free tobacco (u)	0.035	0.000	1.000	1,200,168	0.033	0.000	1.000	1,069,049
Days drank (u)	4.909	0.000	30.000	1,179,159	5.175	0.000	30.000	1,051,527
Current drinker (u)	0.482	0.000	1.000	1,179,159	0.526	0.000	1.000	1,051,527
Avg. Drinks per day (c)	2.295	1.000	76.000	568,406	2.168	1.000	76.000	552,663
Avg. Drinks per day (u)	1.106	0.000	76.000	1,179,159	1.139	0.000	76.000	1,051,527
Binge days last month (c)	4.798	1.000	76.000	155,685	4.468	1.000	76.000	140,175
Any binge (u)	0.132	0.000	1.000	1,179,159	0.133	0.000	1.000	1,051,527
Covariates								
Marital status	0.525	0.000	1.000	1,255,537	0.516	0.000	1.000	1,113,873
High school	0.267	0.000	1.000	1,255,537	0.270	0.000	1.000	1,113,873
Some college	0.285	0.000	1.000	1,255,537	0.273	0.000	1.000	1,113,873
College	0.374	0.000	1.000	1,255,537	0.391	0.000	1.000	1,113,873
Female	0.545	0.000	1.000	1,255,221	0.555	0.000	1.000	1,113,165
Age	54.744	18.000	80.000	1,255,537	55.391	18.000	80.000	1,113,873
White	0.661	0.000	1.000	1,255,537	0.773	0.000	1.000	1,113,873
Black	0.064	0.000	1.000	1,255,537	0.081	0.000	1.000	1,113,873
Hispanic	0.089	0.000	1.000	1,253,577	0.064	0.000	1.000	1,113,586
Unemployment rate	4.120	2.300	13.500	1,253,577	4.475	2.400	9.400	1,113,873
GSP (Billions)	594.110	31.430	3598.103	1,253,577	460.900	35.704	1705.010	1,113,873
PI (Billions)	529.743	31.445	3018.471	1,253,577	396.489	31.646	1340.903	1,113,873
Poverty rate	11.263	5.700	17.100	1,253,577	10.911	6.100	21.400	1,113,873
Governor is democrat	0.284	0.000	1.000	1,253,577	0.431	0.000	1.000	1,113,873
Tobacco tax (cents/pack)	157.732	17.000	320.000	1,255,537	197.041	30.000	435.000	1,113,873
RML	0.172	0.000	1.000	1,255,537	0.172	0.000	1.000	1,113,873
Beer tax ($/gal)	0.412	0.060	1.070	1,255,537	0.208	0.020	1.290	1,113,873
Max observations by treatment group				1,255,537				1,113,873

*Note:* Data from BRFSS and UKCPR, 2016‐2023. Dollar values are in 2020 dollars. Calculations for treated states are for pre‐treatment period. Variables that have (c) represent conditional measures. Variables that have (u) denote undconditional measures.

Abbreviations: GSP: Gross State Product. PI: Personal Income.

**TABLE A2:** Legalized sports betting implementation dates.

State	Any law	Online law	Offline law
Arizona	2021‐09	2021‐09	2021‐09
Arkansas	2019‐07	2022‐03	2019‐07
Colorado	2020‐05	2020‐05	2020‐05
Connecticut	2021‐10	2021‐10	2021‐10
Delaware	2018‐06	2023‐12	2018‐06
District of Columbia	2020‐05	2020‐05	2020‐07
Florida	2023‐12	2023‐12	2023‐12
Illinois	2020‐03	2020‐06	2020‐03
Indiana	2019‐09	2019‐10	2019‐09
Iowa	2019‐08	2019‐08	2019‐08
Kansas	2022‐09	2022‐09	2022‐09
Kentucky	2023‐09	2023‐09	2023‐09
Louisiana	2021‐11	2022‐01	2021‐11
Maine	2023‐11	2023‐11	2023‐11
Maryland	2021‐12	2022‐11	2021‐12
Massachusetts	2023‐01	2023‐03	2023‐01
Michigan	2020‐03	2021‐01	2020‐03
Mississippi	2018‐08	—	2018‐08
Montana	2020‐03	—	2020‐03
Nebraska	2023‐06	—	2023‐06
Nevada	1949‐01	2010‐01	1949‐01
New Hampshire	2019‐12	2019‐12	2020‐08
New Jersey	2018‐06	2018‐08	2018‐06
New Mexico	2018‐10	—	2018‐10
New York	2019‐07	2022‐01	2019‐07
North Carolina	2021‐03	2024‐03	2021‐03
North Dakota	2021‐06	—	2021‐06
Ohio	2023‐01	2023‐01	2023‐01
Oregon	2019‐08	2019‐10	2019‐08
Pennsylvania	2018‐11	2019‐05	2018‐11
Rhode Island	2018‐11	2019‐09	2018‐11
South Dakota	2021‐09	—	2021‐09
Tennessee	2020‐11	2020‐11	—
Vermont	2024‐01	2024‐01	—
Virginia	2021‐01	2021‐01	2022‐07
Washington	2021‐09	—	2021‐09
West Virginia	2018‐08	2018‐08	2018‐08
Wisconsin	2021‐11	—	2021‐11
Wyoming	2021‐09	2021‐09	—

*Note:* Information current as of December 2024. Data compiled by authors based on information from American Gaming Association and various local news reports.

**TABLE A3:** Impact of Online sports betting legalization on Young Men's Binge Drinking: Sensitivity Analyses.

	Binge days	Any binge
	(c)	(u)
Pre‐covid sample:		
Online law	0.2036*	−0.0190
	(0.1192)	(0.0220)
Observations	45,792	141,579
Ever treated states only:		
Online law	0.6232***	−0.0081
	(0.1991)	(0.0114)
Observations	54,864	174,574

*Note:* Standard errors adjusted for clustering around states in parentheses. Coefficients are from weighted regressions with individual level controls (age, marital status, race, ethnicity, and education), state level controls (unemployment rate, gross domestic product, personal income, poverty rate, and governor's party affiliation), and state & year fixed effects. Data is BRFSS 2016‐2019 for pre‐Covid sample and BRFSS 2016‐2023 for ‘ever treated’ sample.

^*^ (*p* < 0.10).

** (*p* < 0.05).

^***^ (*p* < 0.01).

**TABLE A4:** Impact of Online sports betting legalization on Young Men's Binge Drinking: Heterogeneity across demographic groups.

	Binge days	Any binge
	(c)	(u)
White:		
Online law	−0.1098	−0.0033
	(0.1679)	(0.0116)
Observations	172,066	964,599
Black:		
Online law	2.1527***	−0.0053
	(0.4844)	(0.0333)
Observations	11,676	83,551
18–24 year old:		
Online law	−0.0522	−0.0029
	(0.1558)	(0.0077)
Observations	29,047	104,777
25–29 year old:		
Online law	0.4844**	−0.0150
	(0.2216)	(0.0103)
Observations	27,323	81,621
30–34 year old:		
Online law	0.4844**	−0.0150
	(0.2216)	(0.0103)
Observations	26,536	87,638
Married:		
Online law	0.4137	0.0108
	(0.3079)	(0.0174)
Observations	22,417	80,011
Unmarried:		
Online law	0.4056**	−0.0194
	(0.1684)	(0.0121)
Observations	65,871	206,730
College educated:		
Online law	0.3199	−0.0102
	(0.2254)	(0.0112)
Observations	32,331	92,514
Non‐college:		
Online law	0.8159***	−0.0211*
	(0.2477)	(0.0118)
Observations	55,957	194,227

*Note:* Standard errors adjusted for clustering around states in parentheses. Data is BRFSS 2016‐2023.

^*^ (*p* < 0.10).

^**^ (*p* < 0.05).

^***^ (*p* < 0.01).
